# The diagnostic accuracy of deep learning-based AI models in predicting lymph node metastasis in T1 and T2 colorectal cancer: A systematic review and meta-analysis

**DOI:** 10.1097/MD.0000000000045172

**Published:** 2025-11-07

**Authors:** Qihong Guo, Ruiping Wang, Yichen Guo

**Affiliations:** a Department of Radiology, First Hospital of LanZhou University, Lanzhou, Gansu, China; b The First Clinical Medical College of Lanzhou University, Lanzhou City, Gansu, China; c Department of Emergency, First Hospital of LanZhou University, Lanzhou, Gansu, China.

**Keywords:** artificial intelligence, colorectal cancer, lymph node metastasis, meta-analysis

## Abstract

**Background:**

Colorectal cancer (CRC) continues to be a leading cause of cancer-related mortality globally, and accurately predicting lymph node metastasis (LNM) in T1 and T2 lesions is vital for informing treatment strategies. This study aimed to assess the diagnostic accuracy of artificial intelligence (AI)-based models, particularly deep learning (DL) and machine learning (ML) approaches, in predicting LNM risk in CRC.

**Methods:**

A comprehensive literature search was conducted across PubMed, EMBASE, Web of Science, Cochrane Library, and Scopus databases, identifying relevant studies published up to April 6, 2024. A total of 6552 articles were retrieved, and after screening, 12 studies involving 8540 patients were included for qualitative analysis, with 9 studies eligible for quantitative meta-analysis. The methodological quality of the studies was evaluated using the QUADAS-2 tool.

**Results:**

Meta-analysis yielded the following diagnostic parameters: a sensitivity (SEN) of 0.87 (95% CI: 0.76–0.93), specificity (SPE) of 0.69 (95% CI: 0.52–0.82), and AUC of 0.88 (95% CI: 0.84–0.90). The likelihood ratios were 2.80 (95% CI: 1.74–4.50) for positive and 0.18 (95% CI: 0.10–0.34) for negative predictions, with a diagnostic odds ratio of 15.27 (95% CI: 6.49–35.89).

**Conclusion:**

This meta-analysis indicates that AI-based models, particularly DL and ML techniques, demonstrate moderate SEN and good SPE in predicting LNM in T1 and T2 CRC lesions. These findings highlight the potential of AI models as noninvasive diagnostic tools in clinical practice.

## 1. Introduction

Colorectal cancer (CRC) continues to be one of the leading causes of cancer-related mortality globally, despite substantial progress in early detection and treatment strategies.^[1]^ The widespread implementation of population-based screening programs, particularly colonoscopy, has notably enhanced the early detection of CRC, leading to a reduction in overall disease incidence.^[2]^ Following the endoscopic resection of early-stage cancers, a critical challenge arises: determining the necessity for additional radical surgery, a decision that hinges predominantly on the risk of lymph node metastasis (LNM).^[3]^

Traditional diagnostic approaches – such as imaging techniques like magnetic resonance imaging (MRI) and computed tomography (CT), along with histopathological examination – are frequently limited in their ability to accurately gauge LNM risk.^[4]^ For lymph node staging, Magnetic resonance imaging (MRI) demonstrates pooled sensitivity (SEN) and specificity (SPE) of 0.73 (95% confidence interval (CI): 0.68–0.77) and 0.74 (95% CI: 0.68–0.80) respectively, while CT shows optimal SEN of 78.6% and SPE of 75% for detecting metastatic lymph nodes.^[5]^ Histopathological examination, while considered the gold standard, suffers from substantial interobserver variability. For tumor budding assessment, kappa values range between 0.077 and 0.357 (median 0.166) among investigators, and interobserver variability in lymphovascular invasion diagnosis remains substantial even with immunohistochemistry. Additionally, traditional risk stratification models demonstrate modest diagnostic performance, with area under the curve (AUC) values typically ranging from 0.64 to 0.67, highlighting the urgent need for more accurate predictive tools.^[6]^ Key high-risk features, including vascular invasion, tumor budding, and deep submucosal invasion (≥1000 μm), are commonly relied upon as indicators for further intervention.^[7]^ Yet, these evaluations are frequently clouded by interobserver variability and subjective interpretation, leading to inconsistent assessments among pathologists.^[8]^

In recent years, artificial intelligence (AI), particularly machine learning (ML) and deep learning (DL), has emerged as a promising solution to overcome these diagnostic challenges.^[9]^ Real-time applications of AI in colonoscopy have already demonstrated considerable success in polyp detection and classification.^[10]^ These advances suggest potential applications in analyzing more complex features associated with LNM risk, although further validation is needed. The integration of AI with conventional imaging approaches, such as those used in pulmonary nodule assessment, represents another promising direction for improving risk stratification.^[11]^

The growing body of research on AI applications in CRC is compelling, though significant challenges remain, including the need for standardization of assessment criteria, methodological consistency, and robust external validation. Given the urgent clinical need for accurate LNM risk prediction tools and the rapid advancement of AI technologies, a comprehensive systematic review and meta-analysis is essential to synthesize the current evidence on DL-based models for predicting LNM in T1 and T2 CRC. This analysis will provide crucial insights into the diagnostic performance of AI models compared to traditional approaches, identify key factors influencing model accuracy, and establish the current state of evidence to guide future research directions and clinical implementation strategies. By consolidating available data from multiple studies, this meta-analysis aims to determine whether AI-based models can reliably support clinical decision-making in CRC management and identify the challenges that must be addressed before widespread clinical adoption.

## 2. Materials and methods

### 2.1. Literature search and quality assessment

The original study protocol was registered with PROSPERO prior to initiation of the systematic search as an a priori study design(CRD42024607756). This study is a secondary analysis of published data; according to our institutional policy and preferred reporting items for systematic reviews and meta-analyses guidance, ethics approval was waived.^[12]^

This study conducted a comprehensive literature search across multiple databases, including PubMed, EMBASE, Web of Science, Cochrane Library, and Scopus, to identify relevant studies published in English from each database’s inception up to April 6, 2024. The search strategy utilized both medical subject headings (MeSH) and free-text terms. In PubMed, the following search terms were applied: ((Colorectal Neoplasms [MeSH Major Topic]) OR ((Colorectal Neoplasms [Title/Abstract]) OR (Colorectal [Title/Abstract])) OR (((rectum [Title/Abstract]) OR (colon [Title/Abstract])) OR ((rectum [MeSH Major Topic]) OR (colon [MeSH Major Topic])))) AND (DL [Title/Abstract]). The preferred reporting items for systematic reviews and meta-analyses (PRISMA) guidelines were followed for study selection and reporting.

The methodological quality of each included study was evaluated using the Quality Assessment of Diagnostic Accuracy Studies (QUADAS-2) tool to assess bias and applicability in 4 domains: patient selection, index test, reference standard, and flow and timing.^[13]^

### 2.2. Inclusion and exclusion criteria

To ensure relevant and high-quality studies, the following inclusion criteria were established:

Population: Studies involving patients diagnosed with T1 and/or T2 CRC using histopathology as the reference standard.

Intervention: Studies evaluating DL models for predicting LNM in T1 CRC.

Outcomes: Studies with sufficient raw data or other diagnostic accuracy estimates available, such as true positive, false positive, false negative, and true negative values, or estimates of SEN and SPE.

The exclusion criteria were as follows:

1. Duplicate articles.
2. Studies that were not directly related to CRC, such as animal experiments, in vitro research, meta-analyses, reviews, conference abstracts, book chapters, surveys, editorials, and case reports.
3. Studies that did not provide comprehensive data.
4. Studies for which full-text access was not available.

### 2.3. Data extraction

Data from all included studies were independently extracted by 2 researchers, covering details such as the author(s), publication year, country of origin, study design, source of the sample, type of DL model, presence of a control group (if applicable), threshold values, and sample size. Diagnostic accuracy metrics, including true positives, true negatives, false positives, and false negatives, were either directly obtained or computed based on the reported SEN and SPE. Any disagreements between the researchers were resolved through discussion and mutual agreement.

### 2.4. Statistical analysis

A mixed-effects model, implemented with the lme4 package in R, was utilized to integrate diagnostic accuracy data.^[14]^ Key diagnostic accuracy estimates included SEN, SPE, positive likelihood ratio, negative likelihood ratio, and diagnostic odds ratio (DOR), with 95% CIs. The area under the summary receiver operating characteristic (SROC) curve (AUC) served as the overall metric for assessing test performance.

Univariate regression analysis was performed to explore the potential relationships between different study characteristics and diagnostic accuracy outcomes. This approach helped to assess whether specific factors influenced the performance of the DL models in predicting LNM.

Spearman correlation coefficient was employed to examine threshold effects, and heterogeneity was evaluated using the *I*^2^ statistic. Statistical analyses were performed using R (v4.1.0) for mixed-effects modeling and Stata 15.1 for diagnostic accuracy metrics. Statistical significance was set at a threshold of α = 0.05.

## 3. Results

### 3.1. Literature screening

**Figure 1.**
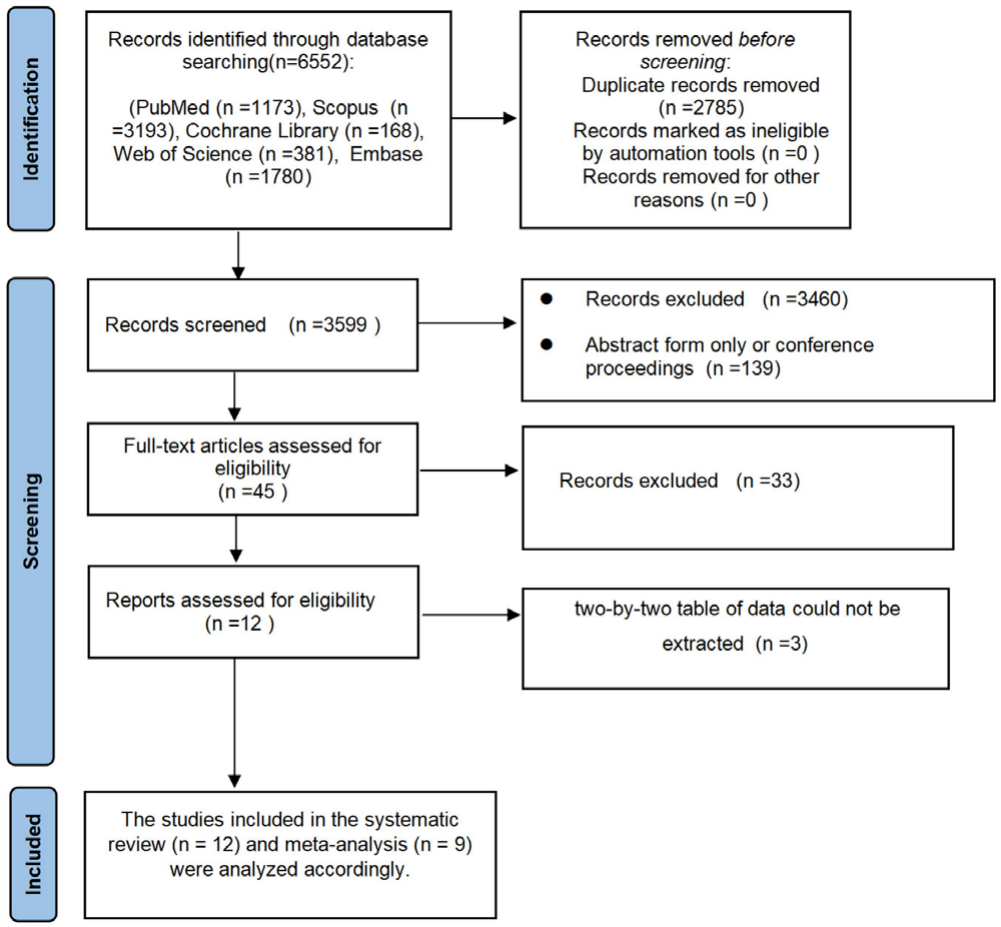
Flowchart of the study selection process.

In this study, a total of 6552 articles were retrieved from multiple databases. After removing 2785 duplicate records, 45 articles were selected for full-text review. Following a thorough assessment, 12 studies were included for qualitative analysis,^[15–24]^ and 9 of these were eligible for quantitative synthesis through meta-analysis.^[16–24]^ A flowchart illustrating the literature screening process is presented in Figure 1, which shows the systematic identification, screening, eligibility assessment, and final inclusion of studies following PRISMA guidelines.

### 3.2. Basic characteristics and quality assessment

The study included 12 articles for qualitative evaluation, with a total of 8540 patients. Of these, 9 studies were eligible for quantitative synthesis through meta-analysis. The data extracted from these individual studies are summarized in Table 1.

**Table 1:** Characteristics of the included studies.

Authors	Year	TP	FP	FN	TN	Data type	Geographical location	Analysis factors
Song et al^[22]^	2022	27	29	0	24	Validation set	South Korea	Pathological
Takamatsu et al^[25]^	2022	–	–	–	–	–	Japan	Non-pathological
Takashina et al^[23]^	2023	15	64	0	21	Validation set	Japan	Pathological
Brockmoeller et al^[18]^	2022 pT1	26	107	7	63	Training set	Denmark	Pathological
	2022 pT2	46	107	11	147	Training set	Denmark	Pathological
Kwak et al^[26^^]^	2020	–	–	–	–	–	Japan	Pathological
Ichimasa et al^[20]^	2022	27	9	1	63	Validation set	Japan	Pathological
Kang et al^[24]^	2020	25	36	16	239	Aggregate dataset	Korea	Pathological
Kasahara et al^[15^^]^	2022	–	–	–	–	–	Japan	Pathological
Kudo et al^[17^^]^	2020	252	994	67	1821	Training set	Japan	Non-pathological
Takamatsu et al^[21]^	2019	19	14	5	239	Training set	Japan	Non-pathological
Ichimasa et al^[16^^]^	2018	9	31	0	60	Validation set	Japan	Non-pathological
Ahn et al^[19]^	2021	1632	424	304	1995	Training set	Korea	Non-pathological

“–“ = not mentioned, FN = false negative, FP = false positive, TN = true negative, TP = true positive.

**Figure 2.**
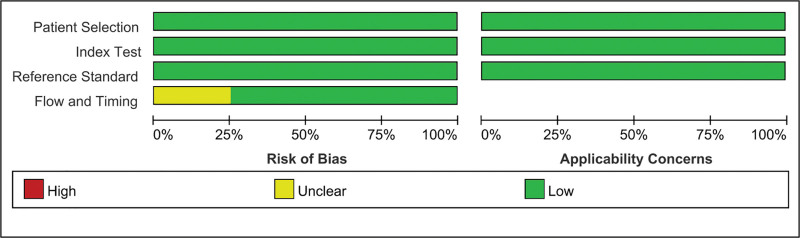
Quality of the included studies scored against the QUADAS-2 criteria. QUADAS-2 = Quality Assessment of Diagnostic Accuracy Studies.

Results for the QUADAS-2 tool for evaluating individual studies for risk of bias and applicability are included in Figure 2, which presents a comprehensive assessment across 4 key domains: patient selection, index test, reference standard, and flow and timing. Quality assessment of the included studies was conducted using the QUADAS-2 tool, and the studies were rated as high quality.

### 3.3. Meta-analysis results

#### 3.3.1. Meta-analysis results

**Figure 3.**
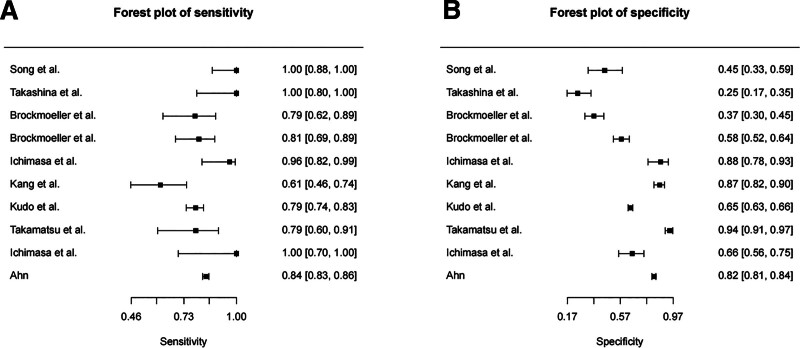
Forest plot of (A) sensitivity and (B) specificity for the prediction of lymph node metastasis (Brockmoeller et al^[18]^ conducted separate analyses for T1 and T2 subgroups, which are presented as individual entries in the meta-analysis.).

**Figure 4.**
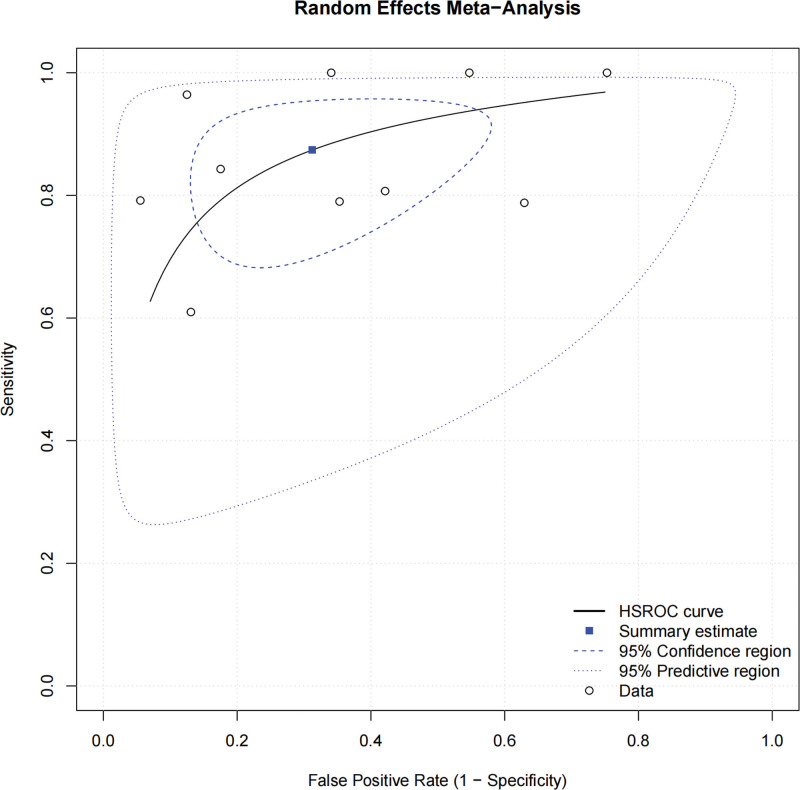
SROC curves for the prediction of lymph node metastasis. SROC = summary receiver operating characteristic.

The Meta-analysis results for the prediction of LNM (Note: Brockmoeller et al^[18]^ contributed 2 separate analyses for T1 and T2 subgroups, resulting in 10 individual data points from 9 studies) were as follows: SEN_combination_ = 0.874 (95% CI: 0.763–0.93), SPE_combination_ = 0.687 (95% CI: 0.515–0.82), PLR_combination_ = 2.797 (95% CI: 1.737–4.503), NLR_combination_ = 0.183 (95% CI: 0.097–0.344), DOR_combination_ = 15.266 (95% CI: 6.493–35.893); SEN_combination_ and SPE_combination_ are shown in Figure 3, which displays forest plots of SEN and SPE estimates with 95% CIs for each included study. The SROC curve, illustrated in Figure 4, demonstrated an AUC of 0.88 (95% CI: 0.84–0.90). This curve plots the relationship between SEN and SPE across all studies, with the summary point representing the overall diagnostic performance.

#### 3.3.2. Univariate meta-regression analysis

**Figure 5.**
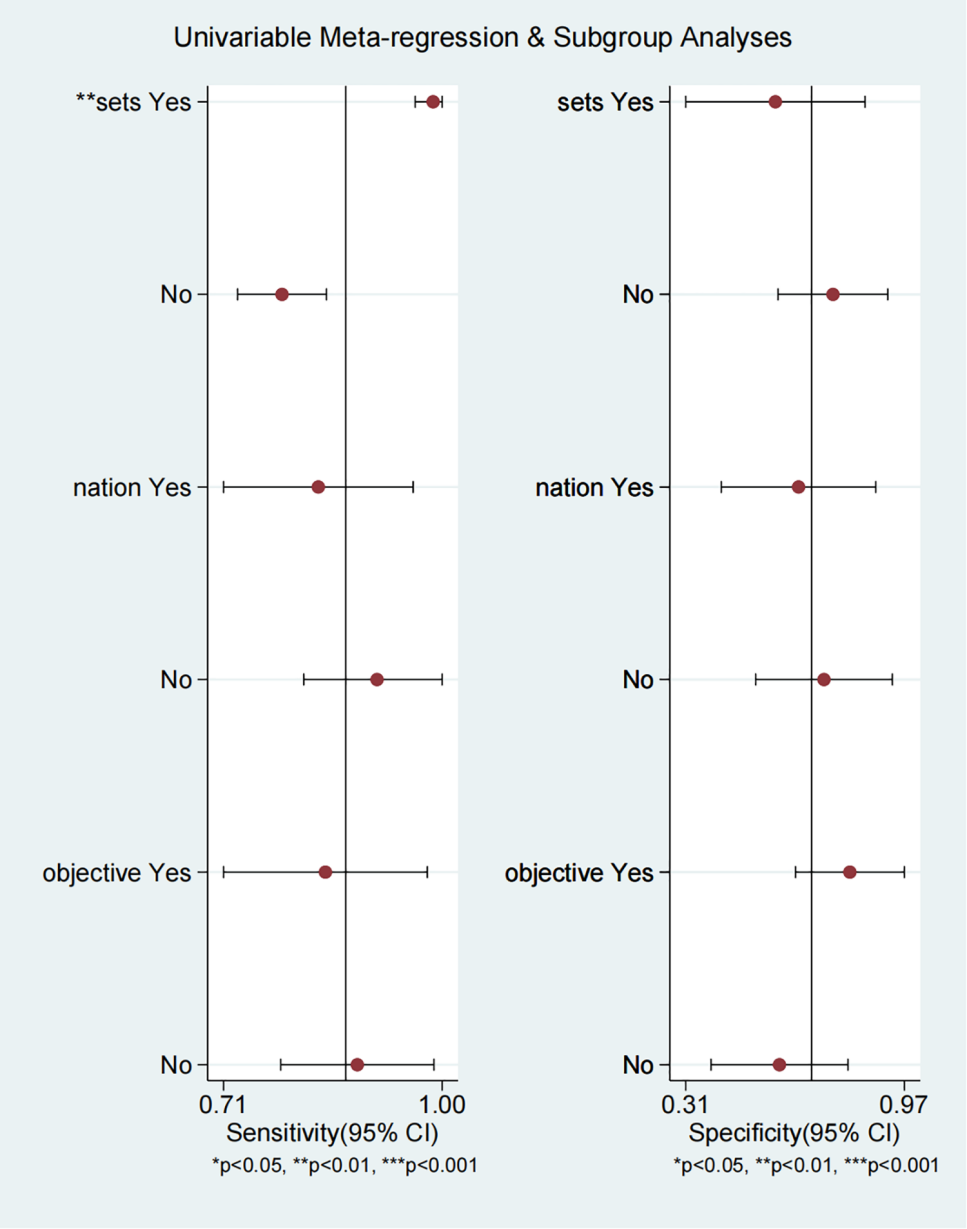
Forest plot of the univariate regression analysis.

The results of the univariate meta-regression analysis, which evaluated the effects of different covariates (training and validation sets, geographical region, and objective) on SEN and SPE, are summarized in Table 2. These covariates were analyzed to assess their impact on the diagnostic performance of DL-based AI models. The results of the univariate regression analysis are shown in Figure 5, which presents the impact of different covariates (training/validation sets, geographical region, and study objectives) on model SEN and SPE through forest plots.

**Table 2:** Results of the univariate meta-regression analysis.

Parameter	Category	Number	Sensitivity [95% CI]	p1	Specificity [95% CI]	p2	LRTChi^2^	*P* value	*I* ^2^	*I*^2^lo	*I*^2^hi
Training and validation sets	Yes	4	0.99 [0.96–1.00]	0	0.58 [0.31–0.85]	.22	18.01	0	89	78	100
Training and validation sets	No	6	0.79 [0.73–0.85]		0.75 [0.59–0.92]						
Geographical region	Yes	5	0.84 [0.71–0.96]	.16	0.65 [0.42–0.88]	.48	2.25	.33	11	0	100
Geographical region	No	5	0.92 [0.82–1.00]		0.72 [0.52–0.93]						
Objective	Yes	4	0.85 [0.71–0.98]	.27	0.80 [0.64–0.97]	.39	2.07	.36	3	0	100
Objective	No	6	0.89 [0.79–0.99]		0.59 [0.39–0.80]						

CI = confidence interval.

When comparing studies using validation sets (Yes) to those using training sets (No), models demonstrated higher SEN in validation sets (0.99, 95% CI: 0.96–1.00) than in training sets (0.79, 95% CI: 0.73–0.85). However, SPE was higher in training sets (0.75, 95% CI: 0.59–0.92) compared to validation sets (0.58, 95% CI: 0.31–0.85). The likelihood ratio test (LRT) for sets showed significant heterogeneity (LRTChi^2^ = 18.01, *P* < .001), with an *I*^2^ value of 89%, indicating substantial between-study variability for this covariate.

The models trained on datasets from non-Japanese populations exhibited slightly lower SEN (0.84, 95% CI: 0.71–0.96) compared to those trained on Japanese datasets (0.92, 95% CI: 0.82–1.00). SPE, however, was comparable between non-Japanese (0.65, 95% CI: 0.42–0.88) and Japanese datasets (0.72, 95% CI: 0.52–0.93). The heterogeneity for this covariate was low, with LRTChi^2^ = 2.25 (*P* = .33) and *I*^2^ = 11%, suggesting minimal between-study variability.

For models applied to non-pathological objectives, SEN was 0.85 (95% CI: 0.71–0.98), slightly lower than for pathological objectives, which yielded a SEN of 0.89 (95% CI: 0.79–0.99). SPE was higher for non-pathological objectives (0.80, 95% CI: 0.64–0.97) compared to pathological objectives (0.59, 95% CI: 0.39–0.80). Heterogeneity for this covariate was negligible, with LRTChi^2^ = 2.07 (*P* = .36) and *I*^2^ = 3%.

The diagnostic accuracy of DL-based AI models varied across the covariates analyzed. Models demonstrated high SEN across all covariates, particularly in validation sets, while SPE showed greater variability, especially when comparing validation vs training sets.

The significant heterogeneity observed for the “sets” covariate suggests that differences between training and validation datasets may influence the diagnostic performance of AI models. In contrast, the covariates “nation” and “objective” exhibited low heterogeneity, indicating more consistent diagnostic performance across these factors.

#### 3.3.3. Sensitivity analysis (excluding largest sample study)

In this SEN analysis, we compared the estimated values and their 95% CIs for SEN and SPE, excluding the study by Ahn (2021) due to its significantly larger sample size. The results are as follows: The estimated SEN was 0.839 (95% CI: 0.736–0.907) without Ahn study, while the original data (including Ahn) had a SEN of 0.874 (95% CI: 0.763–0.938). The CIs for SEN (0.736–0.907 vs 0.763–0.938) still overlap, indicating no statistically significant difference between the SEN estimates, even with the exclusion of the largest study. The estimated SPE was 0.614 (95% CI: 0.395–0.795) without Ahn study, compared to the original SPE of 0.687 (95% CI: 0.515–0.82) including Ahn. The CIs for SPE (0.395–0.795 vs 0.515–0.82) also overlap, suggesting no statistically significant difference between the SPE estimates with or without Ahn study. In summary, excluding the largest study (Ahn, 2021) did not result in statistically significant differences in SEN or SPE estimates when compared to the original data. Table 3 presents the detailed comparison between the primary and SEN analyses.

**Table 3:** Results of comparison between the primary and sensitivity analyses.

Primary analysis				Sensitivity analysis			
Parameter	Estimate	2.5% CI	97.5% CI	Estimate	2.5% CI	97.5% CI	*P* value
Sensitivity	0.874	0.763	0.938	0.839	0.736	0.907	.3927
Specificity	0.687	0.515	0.82	0.614	0.395	0.795	.3927
False positive rate	0.313	0.18	0.485	0.386	0.205	0.605	.3927
False negative rate	0.126	0.062	0.237	0.161	0.093	0.264	.2814
Diagnostic odds ratio	15.266	6.493	35.894	8.328	3.439	20.168	.2156
Likelihood ratio +ve	2.797	1.737	4.503	2.177	1.294	3.662	.2973
Likelihood ratio −ve	0.183	0.097	0.344	0.261	0.156	0.437	.2442
Logit (sensitivity)	1.937	1.167	2.708	1.654	1.027	2.281	.2814
Logit (specificity)	0.788	0.058	1.518	0.466	-0.427	1.358	.3927

CI = confidence interval.

## 4. Discussion

This systematic review and meta-analysis consolidates the most comprehensive and up-to-date evidence regarding the diagnostic performance of DL-based AI models in predicting LNM in T1 and T2 CRC. By synthesizing pooled diagnostic metrics, quality assessments, recent advancements in DL methodologies, and the impact of key covariates, this study offers profound insights into the present state and future trajectories of AI-driven diagnostics in oncology. The findings underscore the remarkable potential of DL models to revolutionize clinical decision-making in CRC management, particularly in the context of LNM risk prediction.

The AUC, often heralded as the gold standard in diagnostic accuracy, stands as a testament to the prowess of AI in this domain. With an AUC of 0.88 (95% CI: 0.84–0.90), our results definitively outshine the conventional models. To put this in perspective, the prediction models from the American Society for Gastrointestinal Endoscopy/European Society of Gastrointestinal Endoscopy (ASGE/ESGE) and the Japanese Society for Cancer of the Colon and Rectum (JSCCR) present AUC values of just 0.67 (95% CI: 0.60–0.74) and 0.64 (95% CI: 0.58–0.70), respectively.^[7]^ These figures not only fall short of our findings but also highlight a glaring gap in diagnostic performance that DL-based AI seamlessly bridges. This stark contrast underscores a fundamental shift in our approach to cancer diagnostics, where the ability to decipher intricate data patterns now outweighs traditional heuristic methods.

The methodological quality of the included studies was largely commendable. Most studies exhibited low risks of bias in key domains, including patient selection, reference standards, and index tests. However, the flow and timing domain revealed an elevated risk of bias, likely attributed to discrepancies in data collection timings, attrition rates, and the differences between training and real-world datasets. Such factors introduce the potential for bias, highlighting the critical need for meticulous data management practices in AI-based research to ensure the reliability and reproducibility of findings. Despite these challenges, the overall applicability of the studies was reassuringly minimal, suggesting that the results are broadly applicable to clinical practice.

The rapid advancements in DL, particularly through CNNs, have revolutionized the analysis of histopathological and imaging data in oncology.^[27]^ DL models have consistently outperformed traditional diagnostic methods, with reported AUC values spanning from 0.75 to 0.99 in previous studies.^[28,29]^ CNNs excel in extracting intricate, high-dimensional features from whole-slide images, allowing for consistent, reproducible diagnoses that surpass human capabilities in terms of accuracy and efficiency.^[26]^ Recent breakthroughs, such as the integration of attention mechanisms and hybrid models that combine CNNs with random forest classifiers, have further refined diagnostic precision, offering a more interpretable and robust approach to LNM prediction.^[30,31]^

Attention-enhanced CNNs, for example, have demonstrated remarkable efficacy by honing in on diagnostically significant regions of pathological images, effectively reducing noise and enhancing diagnostic accuracy.^[32,33]^ Additionally, hybrid approaches that merge image-derived features with clinical data have achieved AUC values as high as 0.94, underscoring their potential to support nuanced risk stratification and individualized treatment decisions.^[34,35]^ However, despite these innovations, challenges persist, including issues related to model explainability, computational cost, and the risk of overfitting, especially in the context of limited datasets. These hurdles must be addressed to further optimize the utility of DL-based models.

The univariate meta-regression analysis revealed several factors that significantly influence model performance. Notably, models tested on validation datasets exhibited markedly higher SEN (0.99, 95% CI: 0.96–1.00) but lower SPE (0.58, 95% CI: 0.31–0.85) compared to those evaluated on training datasets (SEN: 0.79, 95% CI: 0.73–0.85; SPE: 0.75, 95% CI: 0.59–0.92). This discrepancy points to potential overfitting in training datasets, emphasizing the difficulty of generalizing models to external datasets. The significant heterogeneity observed (LRTChi^2^ = 18.01, *P* < .001, *I*^2^ = 89%) further underscores the necessity for robust external validation to ensure the broader applicability of these models.

Our findings are consistent with, yet distinct from, recent systematic reviews in the field. Chen et al^[36]^ conducted a systematic review on AI-based imaging data for predicting distant metastasis in CRC, reporting pooled SEN, SPE, and AUC of 0.86, 0.82, and 0.91, respectively. However, their study included radiomics-based models alongside DL approaches and focused on distant metastasis rather than LNM specifically. In contrast, our meta-analysis exclusively evaluated DL-based models for LNM prediction, providing more targeted evidence for this specific clinical application. Thompson et al^[37]^ performed a qualitative systematic review of AI models for predicting LNM in early-stage CRCs, reporting AUC values ranging from 0.74 to 0.9993. While their review provided valuable insights into model diversity, it did not perform quantitative meta-analysis to provide pooled diagnostic accuracy estimates. Our study addresses this gap by conducting rigorous quantitative synthesis with pooled AUC of 0.88 (95% CI: 0.84–0.90). Baek et al^[38]^ validated AI models specifically for T1 CRC, achieving AUROC values of 0.673 to 0.679, which outperformed traditional Japanese guidelines (AUROC = 0.525). However, their study was limited to T1 cancers only, whereas our meta-analysis encompasses both T1 and T2 CRCs, providing broader clinical applicability. These comparisons highlight that our study provides the most comprehensive quantitative evidence specifically for DL-based LNM prediction across early-stage CRCs.

Geographic factors also played a role, as studies using Japanese datasets exhibited slightly higher SEN (0.92, 95% CI: 0.82–1.00) compared to non-Japanese datasets (0.84, 95% CI: 0.71–0.96), while SPE remained comparable. Low heterogeneity (LRTChi^2^ = 2.25, *P* = .33, *I*^2^ = 11%) suggests minimal geographic variability in diagnostic performance, although the use of diverse, multi-regional datasets is crucial to ensure the global applicability of these models.

Furthermore, non-pathological objectives demonstrated higher SPE (0.80, 95% CI: 0.64–0.97) compared to pathological objectives (0.59, 95% CI: 0.39–0.80), with similar SEN. This observation may reflect the inherent complexity of pathological data, which often exhibit greater variability in histological features. Nevertheless, low heterogeneity (LRTChi^2^ = 2.07, *P* = .36, *I*^2^ = 3%) supports the idea that DL models can be consistently applied across various diagnostic objectives, highlighting their adaptability to diverse clinical scenarios.

To further enhance the clinical utility of DL-based models, several key limitations must be addressed:

1. Dataset size and diversity: To improve model robustness and generalizability, large, high-quality, and diverse datasets are essential.
2. Standardization: Establishing uniform protocols for data collection, preparation, and reporting will mitigate variability across studies.
3. Explainability: The “black-box” nature of DL models remains a significant barrier to clinical adoption. Developing interpretable frameworks, such as attention visualization techniques, will enhance trust and facilitate wider clinical acceptance.
4. External validation: Prospective studies utilizing external datasets are crucial to confirm the clinical efficacy and adaptability of these models in real-world settings.
5. Limited geographic diversity: The majority of studies originated from East Asian populations (Japan and South Korea), which may limit generalizability to other ethnic populations due to potential differences in tumor biology, genetic susceptibility, and histopathological characteristics. Future studies should include more diverse populations to ensure global applicability.

## 5. Conclusion

DL-based AI models demonstrate robust diagnostic performance in predicting LNM in T1 and T2 CRC. Their ability to process complex histopathological and imaging data with exceptional precision positions them as transformative tools in oncology diagnostics. However, overcoming challenges related to dataset diversity, model explainability, and external validation will be pivotal in realizing their full clinical potential. By refining existing methodologies and fostering interdisciplinary collaboration, these advanced diagnostic technologies can be fully integrated into routine clinical practice. These advancements ultimately contribute to improved patient outcomes and the development of more tailored treatment strategies.

## Author contributions

**Conceptualization:** Qihong Guo, Yichen Guo.

**Data curation:** Qihong Guo, Yichen Guo.

**Formal analysis:** Qihong Guo, Ruiping Wang, Yichen Guo.

**Methodology:** Qihong Guo, Ruiping Wang.

**Software:** Qihong Guo, Ruiping Wang.

**Writing** – **original draft:** Qihong Guo, Yichen Guo.

**Writing** – **review & editing:** Qihong Guo, Ruiping Wang, Yichen Guo.

## References

[1] Rex DK Boland CR Dominitz JA. Colorectal cancer screening: recommendations for physicians and patients from the U.S. multi-society task force on colorectal cancer. Am J Gastroenterol. 2017;112:1016–30. 28555630 10.1038/ajg.2017.174

[2] Hashiguchi Y Muro K Saito Y; Japanese Society for Cancer of the Colon and Rectum. Japanese society for cancer of the colon and rectum (JSCCR) guidelines 2019 for the treatment of colorectal cancer. Int J Clin Oncol. 2020;25:1–42. 31203527 10.1007/s10147-019-01485-z PMC6946738

[3] Beets-Tan RGH Lambregts DMJ Maas M. Magnetic resonance imaging for clinical management of rectal cancer: updated recommendations from the 2016 European Society of Gastrointestinal and Abdominal Radiology (ESGAR) consensus meeting. Eur Radiol. 2018;28:1465–75. 29043428 10.1007/s00330-017-5026-2 PMC5834554

[4] Ueno H Mochizuki H Hashiguchi Y. Risk factors for an adverse outcome in early invasive colorectal carcinoma. Gastroenterology. 2004;127:385–94. 15300569 10.1053/j.gastro.2004.04.022

[5] Beets-Tan RGH. Rectal cancer: review with emphasis on MR imaging. Radiology. 2007;232:335–46. 10.1148/radiol.2322021326 15286305

[6] Nagendran M Chen Y Lovejoy CA. Artificial intelligence versus clinicians: systematic review of design, reporting standards, and claims of deep learning studies. BMJ. 2020;368:m689. 32213531 10.1136/bmj.m689 PMC7190037

[7] Watanabe T Muro K Ajioka Y; Japanese Society for Cancer of the Colon and Rectum. Japanese society for cancer of the colon and rectum (JSCCR) guidelines 2016 for the treatment of colorectal cancer. Int J Clin Oncol. 2018;23:1–34. 28349281 10.1007/s10147-017-1101-6 PMC5809573

[8] van Putten PG Hol L van Dekken H. Inter-observer variation in the histological diagnosis of polyps in colorectal cancer screening. Histopathology. 2011;58:974–81. 21585430 10.1111/j.1365-2559.2011.03822.x

[9] Kudo SE Mori Y Misawa M. Artificial intelligence and colonoscopy: current status and future perspectives. Dig Endosc. 2019;31:363–71. 30624835 10.1111/den.13340

[10] Mori Y Kudo SE Misawa M. Real-time use of artificial intelligence in identification of diminutive polyps during colonoscopy: a prospective study. Ann Intern Med. 2018;169:357–66. 30105375 10.7326/M18-0249

[11] Kim CH Huh JW Kim HR Kim YJ. Indeterminate pulmonary nodules in colorectal cancer: follow-up guidelines based on a risk predictive model. Ann Surg. 2015;261:1145–52. 25119121 10.1097/SLA.0000000000000853

[12] Page MJ McKenzie JE Bossuyt PM. The PRISMA 2020 statement: an updated guideline for reporting systematic reviews. BMJ. 2021;21:n71. 10.1136/bmj.n71 PMC8005924 33782057

[13] Whiting PF Rutjes AW Westwood ME; QUADAS-2 Group. QUADAS-2: a revised tool for the quality assessment of diagnostic accuracy studies. Ann Intern Med. 2011;155:529–36. 22007046 10.7326/0003-4819-155-8-201110180-00009

[14] Bates D Mächler M Bolker B. Fitting linear mixed-effects models using lme4. J Stat Softw. 2015;67.

[15] Kasahara K Katsumata K Saito A. Artificial intelligence predicts lymph node metastasis or risk of lymph node metastasis in T1 colorectal cancer. Int J Clin Oncol. 2022;27:1570–9. 35908272 10.1007/s10147-022-02209-6

[16] Ichimasa K Kudo SE Mori Y. Artificial intelligence may help in predicting the need for additional surgery after endoscopic resection of T1 colorectal cancer. Endoscopy. 2018;50:230–40. 29272905 10.1055/s-0043-122385

[17] Kudo SE Ichimasa K Villard B. Artificial intelligence system to determine risk of T1 colorectal cancer metastasis to lymph node. Gastroenterology. 2021;160:1075–84.e2. 32979355 10.1053/j.gastro.2020.09.027

[18] Brockmoeller S Echle A Ghaffari Laleh N. Deep learning identifies inflamed fat as a risk factor for lymph node metastasis in early colorectal cancer. J Pathol. 2022;256:269–81. 34738636 10.1002/path.5831

[19] Ahn JH Kwak MS Lee HH. Development of a novel prognostic model for predicting lymph node metastasis in early colorectal cancer: analysis based on the surveillance, epidemiology, and end results database. Front Oncol. 2021;11:614398. 33842317 10.3389/fonc.2021.614398 PMC8029977

[20] Ichimasa K Nakahara K Kudo SE. Novel “resect and analysis” approach for T2 colorectal cancer with use of artificial intelligence. Gastrointest Endosc. 2022;96:665–72.e1. 35500659 10.1016/j.gie.2022.04.1305

[21] Takamatsu M Yamamoto N Kawachi H. Prediction of early colorectal cancer metastasis by machine learning using digital slide images. Comput Methods Programs Biomed. 2019;178:155–61. 31416544 10.1016/j.cmpb.2019.06.022

[22] Song JH Hong Y Kim ER Kim SH Sohn I. Utility of artificial intelligence with deep learning of hematoxylin and eosin-stained whole slide images to predict lymph node metastasis in T1 colorectal cancer using endoscopically resected specimens; prediction of lymph node metastasis in T1 colorectal cancer. J Gastroenterol. 2022;57:654–66. 35802259 10.1007/s00535-022-01894-4

[23] Takashina Y Kudo SE Kouyama Y. Whole slide image-based prediction of lymph node metastasis in T1 colorectal cancer using unsupervised artificial intelligence. Dig Endosc. 2023;35:902–8. 36905308 10.1111/den.14547

[24] Kang J Choi YJ Kim IK. LASSO-based machine learning algorithm for prediction of lymph node metastasis in T1 colorectal cancer. Cancer Res Treat. 2021;53:773–83. 33421980 10.4143/crt.2020.974 PMC8291173

[25] Takamatsu M Yamamoto N Kawachi H. Prediction of lymph node metastasis in early colorectal cancer based on histologic images by artificial intelligence. Sci Rep. 2022;12. 10.1038/s41598-022-07038-1 PMC8863850 35194184

[26] Kwak MS Lee HH Yang JM. Deep convolutional neural network-based lymph node metastasis prediction for colon cancer using histopathological images. Front Oncol. 2021;10. 10.3389/fonc.2020.619803 PMC7838556 33520727

[27] Litjens G Kooi T Bejnordi BE. A survey on deep learning in medical image analysis. Med Image Anal. 2017;42:60–88. 28778026 10.1016/j.media.2017.07.005

[28] Krogue JD Azizi S Tan F. Predicting lymph node metastasis from primary tumor histology and clinicopathologic factors in colorectal cancer using deep learning. Commun Med. 2023;3. 10.1038/s43856-023-00282-0 PMC10125969 37095223

[29] Song JH Kim ER Hong Y. Prediction of lymph node metastasis in T1 colorectal cancer using artificial intelligence with hematoxylin and eosin-stained whole-slide-images of endoscopic and surgical resection specimens. Cancers. 2024;16:1900. 38791978 10.3390/cancers16101900 PMC11119228

[30] Chang X Wang J Zhang G. Predicting colorectal cancer microsatellite instability with a self-attention-enabled convolutional neural network. Cell Rep Med. 2023;4:100914. 36720223 10.1016/j.xcrm.2022.100914 PMC9975100

[31] Merabet A Saighi A Saad H. AI for colon cancer: a focus on classification, detection, and predictive modeling. Int J Med Inf. 2025;206:106115. 10.1016/j.ijmedinf.2025.106115 41075424

[32] Elshamy R Abu-Elnasr O Elhoseny M. Enhancing colorectal cancer histology diagnosis using modified deep neural networks optimizer. Sci Rep. 2024;14. 10.1038/s41598-024-69193-x PMC11341685 39174564

[33] Zhou J Foroughi pour A Deirawan H. Integrative deep learning analysis improves colon adenocarcinoma patient stratification at risk for mortality. eBioMedicine 2023;94:104726. 37499603 10.1016/j.ebiom.2023.104726 PMC10388166

[34] Zhang D Zheng B Xu L. A radiomics-boosted deep-learning for risk assessment of synchronous peritoneal metastasis in colorectal cancer. Insights Imaging. 2024;15. 10.1186/s13244-024-01733-5 PMC11183032 38886244

[35] Ao W Wu S Wang N. Novel deep learning algorithm based MRI radiomics for predicting lymph node metastases in rectal cancer. Sci Rep. 2025;15. 10.1038/s41598-025-96618-y PMC11982536 40204902

[36] Chen L Xu F. Diagnostic accuracy of artificial intelligence based on imaging data for predicting distant metastasis of colorectal cancer: a systematic review and meta-analysis. Front Oncol. 2025;15. 10.3389/fonc.2025.1558915 PMC12104061 40421093

[37] Thompson N Morley-Bunker A McLauchlan J. Use of artificial intelligence for the prediction of lymph node metastases in early-stage colorectal cancer: systematic review. BJS Open. 2024;8. 10.1093/bjsopen/zrae033 PMC11026097 38637299

[38] Baek JE Yi H Hong SW. Artificial intelligence models may aid in predicting lymph node metastasis in patients with T1 colorectal cancer. Gut Liver. 2025;19:69–76. 39778879 10.5009/gnl240273 PMC11736321

